# A Role of Production on E-Commerce and Foreign Policy Influencing One Belt One Road: Mediating Effects of International Relations and International Trade

**DOI:** 10.3389/fpsyg.2021.793383

**Published:** 2022-01-11

**Authors:** Hanxiao Wang, Bei Liu

**Affiliations:** ^1^Business School, Jiangxi University of Science and Technology, Nanchang, China; ^2^Business School, Gongqing College of Nanchang University, Nanchang, China

**Keywords:** foreign policy, international relations, factors of production, international trade, e-commerce, OBOR (One Belt One Road)

## Abstract

One Road One Belt has made a drastic change not only to the lives of people but also to their minds and future prospective. This initiative has connected not only countries but has consolidated trading patterns. It has not only impacted physical trade but has also boosted the e-commerce of China. Therefore, this study has tried to find the major patterns of trading across the globe and digital commerce considering the factors of production. China, being the cheapest country for manufacturing, has excelled in the e-commerce as well. The targeted population for this study was contractors, marketers, logistic service providers, and engineers. The sample size in this study was 329. Data collection was done through a survey developed on the Likert scale. The software used for the data analysis was Smart-PLS for structural equation modeling. Findings of the study show that factors of production and international trade have an impact on e-commerce. Moreover, the foreign policy and international relations have also been found to have a significant role in e-commerce (digital entrepreneurship).

## Introduction

China’s leadership has acknowledged the country’s various problems and has opted to abandon its development plan. This strategy was considered as a long-term sustainable approach. In this approach, China succeeded in developing infrastructure in the country and this proved to be a factor in nearly everything for the whole world. There was a need to transform into a developed nation from a developing country. So, One Belt One Road (OBOR) became a project to link China with other Asian, European, and African nations (Yingfei et al., 2021). This initiative is not a copy of the Marshall plan but a plan that seeks to build infrastructure across different regions. The Asian Infrastructure Investment Bank, established in 2015, is financing OBOR projects among the participating countries. The initiative of One Belt One Road is indeed to resurrect and improve the essence of a historic silk route and achieve the Asian era prophecy. The project comprises a number of mega-projects, but the two most important are the Maritime Silk Road and Economic Belt of Silk Road. These were originally made public in 2013, during trips of Chinese President Xi Jinping to Indonesia and Kazakhstan. The ancient Silk Road has made history, commitments, and advancements. It was an initiative that the Government of China took into consideration during 2013 under the belt and road initiative. It has had a dramatic influence on global commerce, and it also contributed to developing collaborations on a global scale (Hao et al., 2020).

The great initiative of One Belt One Road can only be implemented because of far-reaching successes in trade liberalization, which have been progressively advanced during the last several years due to the consensus on trade and tariffs. Geography plays a part in the OBOR initiative, as does geopolitics (Visvizi et al., 2019). The One Belt One Road program is executed in various ways, including involving specific nations directly or demanding corridor-type coordination with all of its consequences. One Belt One Road is expected to reshape how we think about, conceptualize, and handle production, transportation, and policy, particularly innovation policy, in order to achieve long-term development and growth. In this regard, the so-called developing markets that are part of One Belt One Road will be the most intriguing instances to watch and monitor. Given the far-reaching consequences that the initiative of One Belt One Road is thought to have in terms of limits of business, society, economics, and politics, it becomes crucial to elaborate and ensure that the basic processes which are involved and their linkages which they engender are easily understood (Gabuev, 2016). In the era of contemporary global market commerce, a few academics have proposed the concept of a cross-border e-commerce model of development in the economic zone, which would use e-commerce as an international trade channel, making international trade more convenient, and breaking regional constraints in traditional inbound trade (Wu et al., 2020).

In China’s home market, e-commerce is a prevalent transaction type that utilizes the Internet as a platform and financial services to perform transactions. The pattern and limits of conventional physical transactions in China have completely changed due to this type. Because of the importance of e-commerce, it is worth considering whether e-commerce can be integrated into the One Belt One Road initiative, and the efficiency of international trade forms under One Belt One Road. One of the major benefits of OBOR projects is the fact that they provide a slew of business networks and modes of operations for industries which are in operation among different nations and China. The instance of Hewlett Packard (HP) is an excellent illustration. Hewlett Packard has produced and exported their computers and peripherals to the state of China for a long time. Initially, production plants were mostly located along the shore, such as in Southern China. However, because of rising labor costs and a labor scarcity in the coastal region, HP began to transfer its production facility inland (Wong et al., 2018). Chongqing currently employs 87% of HP’s Chinese manufacturing employees. Today, train connections provide linkage between 34 cities of Europe with 35 cities of China, with enhanced frequency of services. As a result, the OBOR program provides enterprises with additional efficiency and flexibility in terms of shipping routes and modal options. E-commerce has risen in popularity, but e-commerce across borders has significantly grown.

According to different analyses, the cumulative growth rate of transactions across borders could double compared to transactions generally held between 2014 and 2020. Cross-border trade, on the other hand, is fraught with difficulties. As illustrated, cross-border commerce encounters a lot of “logistics friction,” which results in higher prices and longer lead times. Resistance has also been found to be significantly worse in emerging countries than in industrialized economies. The World Bank, for example, discovered that traversing the boundaries of regions of Sub Saharan Africa could be as time-consuming and expensive as crossing the borders of wealthy nations. Pursuing positive interaction, convergence, and synergy across efforts, thereby generating possibilities for collaboration, is one approach that may avoid conflict and reduce stress. The Bangladesh, India, China, and Myanmar project, for example, illustrates how regional efforts may be combined with the OBOR plan to create win-win cooperation (Das, 2017).

Alongside infrastructure, the OBOR initiative advocated for more agreements on online trading and platforms for communication in a white paper during March of 2015 to create an “information silk road.” The proposal’s third pillar, known as the “digital new silk road” includes digital industries including communications, technology of Internet-of-Things (IoT), and digital commerce. The global process of digitization proved to be a strong method for reducing the previously mentioned logistics friction. The results of e-commerce across borders are linked to the foreign policy of China of OBOR. Recently, new national-level regulations were enacted, and specialized “Cross-border E-commerce Zones” were formed among several cities. The e-commerce across borders became a major sector within Shanghai, Fujian, and Guangdong’s national “Free Trade Zones.” China has created 13 “cross-border e-commerce extensive pilot areas” since 2015. Cross-border e-commerce has extended the routes for businesses wanting to access the worldwide market through the Internet and information technology, and has emerged as a critical technical basis for fostering economic integration and trade globalization (Lee and Shen, 2020). According to sources, China has been the world’s largest e-commerce market since 2013. China has also surpassed the United States as the world’s second-largest digital economy since 2016. In 2016, the digital economy generated more than 30% of China’s GDP (74.4 trillion yuan, or $11.2 trillion). By 2021, this figure is anticipated to exceed 35% (Lee and Shen, 2020). Apart from the initiatives taken by the government, private businesses of China showed their keen interest to showcase their association of development with the Silk Road of China worldwide. During 2016 and 2017, the first free zone of digital trading was set up in Malaysia by the Alibaba group. This became possible due to an embodiment of Alibaba founder Jack Ma’s vision which was about providing a platform globally for digital trading (named eWTP), which he proposed during the 2015 G20 meeting in Hangzhou. Because nations along OBOR became the most significant locations for Jack Ma’s growth goals across the globe, the platform for global digital trading is linked to the Digital Silk Road (Lee and Shen, 2020). This possibility to use emerging countries with staggering economy as part and parcel of the value chain globally cannot be introduced without danger (Roblek et al., 2020; Dahwan and Raju, 2021). Without a question, OBOR is a significant, revolutionary initiative that has the potential to significantly influence many people’s lives. Some academics have even compared the Marshall plan to OBOR. It comes with a lot of dangers, but it also offers a lot of benefits. Innovative approaches to manage logistics and supply internationally, utilizing the OBOR principle, can yield significant benefits. Such possibilities are also ideal for logistics and supply experts and we hope to see more of this type of study develop in the near future (Chan, 2018).

China’s increasing importance in international relations has empowered Chinese IR researchers to argue for a “Chinese method” of thinking about international relations and to infuse traditional Chinese notions into mainstream IR studies in recent years. Given China’s rising economic strength and international influence, Qin Yaqing, President of the China Foreign Affairs University, argues that building Chinese IR theory has gained traction since the turn of the century (Qin, 2011). China provides an intriguing case study when it comes to the relationship between international war and international trade. Since its foundation, China has had great and difficult ties with foreign countries, with the latter occurring frequently. China has significantly grown its manufacturing and service exports and imports over the last four decades, establishing itself as a global player with increasing economic might and political clout (Lukin, 2019). According to common thinking, countries with a higher volume of international commerce and a higher degree of economic interdependence are more restrained in international wars. As a result, consumers and companies tend to have less “home biases” in their purchases of goods and services in a calmer and happier atmosphere. While proponents of increased international economic integration have highlighted common wisdom as a means of ensuring global stability.

The factors that could strongly advocate e-commerce practices in China due to OBOR are kept under consideration. Due to promising results shown in the past in e-commerce, we decided to plan a study to look into the impact of OBOR on e-commerce in China. For conducting the research, several factors such as international relations and international trade were kept as mediating factors in the development of China.

## Review of Literature

### Effects of Foreign Policy on International Relations

Foreign policy could be defined as a country’s approach to interact and relate with the environment across borders. It can be defined as improvement and preservation of the security and strengths of the country with economic connections to other countries to maintain the social and economic integrity of the country. While discussing the analysis on foreign affairs, it deals with different organizations, governments of the countries, and the individuals involved. It also analyzes how they work together to interact under the international systems. Resultantly, policy on foreign affairs may also be narrated as usage of different resources such as sanctions, diplomatic avenues, and innovative media for the structuring of politics both at a local and foreign level and development of such an atmosphere that suits the goals of the country. It has long been debated that international-level relations are the major constituents of a foreign policy. Foreign policy upon analysis provides the understanding of decisions taken individually or collectively on a group level because most decisions have a direct impact on or directly influence external factors. All this can have an impact on local entities due to their linkage with the entities of external factors (Hudson and Day, 2020). It has long been thought that working for linkage between international relations and a country’s foreign policy is advantageous (Smirnova and Rudenko, 2017; Simangunsong et al., 2019; MacDonald and Hasmath, 2020).

International affairs on foreign policy provide a notable stage in the culture of countries these days. As countries have developed their own foreign policies and they take decisions on the basis of their policies and they have demonstrated consequences in the long and short run, it becomes crucial for contacts to collaborate with other countries. During World War I, analyzing a country’s foreign policy was given a lot of weight (Alden and Aran, 2016). A big challenge or goal of foreign policy would be to keep acknowledging intuitive knowledge over explicit knowledge, explicit knowledge provides the opportunity to reexamine the basis of questions and the assumptions based on the learnings of the lessons from past experience, allowing us to compare and contrast things in a systematic way. Experts have described the word “foreign policy” in a variety of ways, but they all agree that it refers to a country’s behavior toward other countries. For example, Hermann described foreign policy as “the distinct intentional action that comes from a person or group of persons’ political level choice.” Keeping in view the literature available on foreign policy and international relations, one can safely say that foreign policy plays an important role in determining international relations. A country’s relations with its counter states define the development and progress in the economy, therefore we assumed that foreign policy would have a certain impact on international relations. So, to test the assumption, the following hypothesis was formulated.

*H_1_: Foreign policy has a positive effect on international relations*

### Effects of Foreign Policy on E-Commerce

E-commerce has flourished throughout the world as the Internet has grown and digital technology has advanced. E-commerce is a type of business where buyers order and pay for items online, and vendors deliver them either in the physical or electronic form (Bergendahl, 2005). E-commerce across border refers to the foreign policy of China for international commercial operations in which people or businesses from other nations use e-commerce portals for trading and settlement transactions and depend on the logistics service system that provides products (Cui et al., 2019). Since 2008, China’s cross-border e-commerce sector has been on the rise. Since the adoption of marketing strategies and the building of e-commerce platforms as part of the One Belt One Road project in 2013, the sector has grown quickly. With an average yearly growth rate of over 20%, China’s total cross-border e-commerce transaction volume grew from 0.7 trillion yuan in 2008 to 10.5 trillion yuan in 2019. Simultaneously, cross-border e-commerce transactions accounted for even more than 30% of overall exports and imports in 2019, up from less than 5% in 2008.

Foreign policy’s economic relevance in cross-border digital commerce has recently gotten a lot of attention. Cross-border e-commerce helps international trade, according to some of the relevant literature (Giuffrida et al., 2017). Others looked at the effect of cross-border e-commerce on a company’s export growth (Houghton and Winklhofer, 2004). Cross-border e-commerce has been relatively easy for foreign policy makers of China due to its value addition to the GDP of China. It has been the top initiative for devising and modifying foreign policy addressing the benefits of e-commerce (Wang and Wang, 2018). Hence, going through the literature, it was assumed that foreign policy would impact e-commerce growth and maximum returns. So, the following hypothesis was formulated.

*H_2_: Foreign policy has a positive effect on e-commerce*

### Impact of Factors of Production on International Trade

There are several factors of production that should be kept in focus while devising a trading strategy in any business. Land is one of the most important environmental factors which is involved and should be kept under consideration while developing any business. Businesses directly deal with international trades, and over time, land utilization has impacted deforestation and higher CO_2_ emissions. The decontamination of water can be implemented through several methods but the impact caused by deforestation can only be reversed by afforestation. The use of efficient land in trade, like developing international trade zones on land which is not directly or indirectly impacting the environment, is the need of the hour (Liu et al., 2014). It is worth mentioning that trade at any level provides opportunities for jobs and employment for skilled and unskilled laborers side by side. International trading opportunities in the commerce sector and job opportunities have always had a tumultuous connection. Conventionally, economists associated with trading have focused on improving the impacts of trading internationally through efficiency management, producing a slight influence on the employment available, for medium to longer durations. Politicians, policy makers, and the officials associated with governments say that there is a positive impact of international relations with creation and opportunities of employment. They claim that many jobs are produced resultantly with improved exports. Current trade simulations, which include certain labor market frictions, suggest that trade liberalization may impact relative unemployment between various categories of labor in a number of ways. Furthermore, these models conclude that international commerce can have a beneficial or negative impact on an economy’s total unemployment rate (Mohler et al., 2018).

The investors across the globe must answer two basically interconnected questions: where and what to spend and in what quantities and under which sections? Due to their high economic development and the return ability of specific local businesses or industries, some nations might be appealing through an equity standpoint. Other nations may be more appealing from a fixed income standpoint due to their low interest rates and price stability. Traders must examine various country variation characteristics including projected gross domestic product, financial policies, trading and monetary policies, and competitive ability to find areas that are likely to provide attractive investment possibilities. For failing businesses, exporting may be a realistic option. However, the various processes and laws that must be addressed before exporting may be enough to deter businesses from doing so. This paper explores the aforementioned roadblocks and offers a checklist to make the process of exporting a product to a foreign market go more smoothly, especially when entrepreneurs have been confronted by host company environments that are fundamentally different from their home nations as a result of international trade operations.

Despite decades of entrepreneurship study, we know relatively little about these entrepreneurs and their strategic behavior in developing and maintaining multinational enterprises. This viewpoint contends that considerable differences in home country institutional frameworks explain differences in potential transnational entrepreneurs’ entrepreneurial endowments. Transnational entrepreneurship is rooted in transnational actor networks that enable cross-border commercial activities to be successful (Yeung, 2002). Keeping in view the impact of factors of production on international trade, the following hypothesis was developed to check the significance of the study.

*H_3_: Factors of production have a positive effect on international trade*

### Impact of Factors of Production on E-Commerce

In traditional commerce, factors of production require land, labor, capital, and entrepreneurship for running a business. With the changing dimensions of commerce toward e-commerce, their utilization has also been changed and more oriented toward online and digital commerce. Companies like Amazon and Walmart are altering the entire nature of local retail in their quest to deliver items directly to consumers at an ever-increasing rate and speed. This ongoing “fulfillment” transformation will continue to play a key role in planning and land use. Many malls and brick-and-mortar businesses are said to have closed as a result of the development of e-commerce. Some of these shopping malls are being transformed into e-commerce “fulfillment” facilities. One recent example is Amazon’s purchase of the Randall Park Mall in Ohio, which was formerly regarded as one of the country’s largest retail malls. This paper looks at how e-commerce might affect land usage and the built environment.

Timing is crucial in the e-commerce industry, which emphasizes the necessity of fulfillment center locations. The site must take supply chain reliability into account, particularly for imported items. As a result, proximity to seaports, airports, and inland ports is critical to ensure that commodities are transferred quickly and efficiently from ships, railways, and vehicles to distribution facilities. As a result, the optimum strategy is in regional centers, where closeness to air and land shipping allows for quick delivery while also lowering shipping costs. Consumers also believe that home delivery is the greatest choice, according to an e-consumer study. In 2015, most orders were delivered through click and collect, which means that deliveries were made to pick-up sites, despite the fact that most buyers preferred home delivery. Similarly, labor or employment also has an impact on e-commerce and that depends on the availability and skill. Many people anticipated that the rise of e-commerce would wreak havoc on the labor market, particularly in the retail sector. However, it resulted in the creation of jobs in a variety of fields. Many examples are shown in digital dividend payments of how e-commerce increases job prospects for individuals who may have previously been excluded from the global market place. The Internet, according the research, allows many small businesses to participate in global commerce, resulting in greater inclusion. China is the country that has benefited the most from it in terms of development. According to China’s State Information Center, the country’s recent e-commerce boom has produced 10 million jobs in online retailers and related services, accounting for roughly 1.3% of total employment. In the economy, new options for entrepreneurship and self-employment are quickly expanding.

Customer happiness and revenue development tend to go hand in hand in any organization. The happier the consumers are, the more items you sell, and the better your bottom line will be. However, everyone who has worked in the e-commerce industry understands that it is not that straightforward. Unexpected costs, new obligations, and cash flow problems can stifle or even halt your expansion. While all businesses have problems with working capital, e-commerce businesses have considerably fewer finance alternatives than their brick-and-mortar competitors. E-business innovations are a type of digital transformation of company operations that significantly impact current business practices. Furthermore, accepting change in company culture, which has been broadly defined as a system of shared meaning inside an organization that determines how people act, is at the heart of business innovations. Organizational e-business is connected to the Internet and the increased usage and use of computers (Sofyani et al., 2020). Keeping in view the literature on factors of production, the following hypothesis was developed to check the impact on e-commerce.

*H_4_: Factors of production have a positive effect on e-commerce*

### Role of International Relations in E-Commerce

It is widely understood that international relations are like a backbone for any country in this global environment. International relations offer so much for the countries’ security, sustainability, economies, and stability. Good relations with super economies offer a lot of opportunities for international trade including goods, logistics, sourcing, production, etc. In the scenario of COVID-19 and the post pandemic situation, most communities have shifted their trade trends toward online activity-based trading. So, they have developed infrastructure which suits their e-commerce positively. The conventional approach is being replaced by the modern digital approach for e-commerce. The thing which remains the same is the relations of a country with other countries. If one is interested in trading with other country, they have to have good relations with them. This has ignited competition among super economies and is impacting smaller economies which depend on international trade (Balcerzak and Bernard, 2017). Countries that are more dependent on China-based products and trade are more beneficial as they have good international relations with China and ally countries. Countries that mostly use United States-based products and services for trading are still lagging due to COVID-19 interventions. China is progressing well in these difficult times. To keep up with growth, the United States is also working hard on strengthening their ties with other countries, providing them great e-commerce-related opportunities. Digital commerce provides the opportunity for both developing and developed countries with new improved potential (Ubaydullaev, 2021). Advantages will come from targeting rich countries in shorter terms, while developing nations will benefit in longer durations. In the short term, developing countries have less or unimproved infrastructure which is essential to properly utilize the benefits of the Internet.

However, in longer durations, countries will certainly benefit in the development of infrastructure related to information technology which is necessary in order to initiate development (Kondratiuk-Nierodzińska, 2016). The economic benefits of digital commerce are placed into three diverse categories, including productivity, pricing, and companies. Companies have been forced to evaluate and adapt their supply chain strategy due to a mix of technology and commercial pressures. Firms have sought more coordination and collaboration across supply chain partners to wring out inefficiencies that may occur inside business transactions to remain competitive. Many of the activities may be completed outside of the company, using electronic markets.

As a result, the Internet and its services have aided in improving supply management efficiency. The countries with good international relations with other countries will consider this and help each other in providing the pillars to e-commerce of the participants. The web is substantially boosting digital commerce across border prospects for industry-to-industry and industry-to-consumer transactions. The Internet, in particular for business-to-consumer transactions, provides opportunities for a potential revolution in commerce globally; the individualization of trade. It provides the opportunity for customers to trade with vendors of foreign origin directly without going to the country of a seller. The Internet has allowed retailers to place their storefronts in front of consumers across the globe in the form of web pages.

The consumer market has grown to unprecedented proportions as a result of technological advancements (Terzi, 2011). The importance of e-commerce growth may be measured in numbers. Global e-commerce was valued at more than $150 billion in 1999. Around 80% of such transactions were between two businesses. Even though the United States and Canada dominate the globe in e-commerce expenditure, other nations are rapidly purchasing online. Global e-commerce expenditure was expected to grow by more than 90% by 2014. All the literature available suggests that international relations have a lot to do with e-commerce and have a certain effect on e-commerce. So, to evaluate the perspective, the following hypothesis was devised.

*H_5_: International relations have a positive effect on e-commerce*

### Impact of International Trade on E-Commerce

It is widely known that custom rituals of trade between countries have been in practice before the invention of e-commerce activities. After the onset of e-commerce, several ease-at-work activities came in to being. These commerce activities suited both the customers and sellers. E-commerce changed the dimensions of trading and commerce of the regions. Firstly, trade primarily occurred from companies to companies but it changed due to production in some countries, logistics in some other countries, and trading in some other regions. All this happened due to the prevalence of e-commerce across the globe or across borders. This has impacted international trading and ultimately, international trading itself has impacted e-commerce. International trading has provided dimensions for e-commerce. All countries gain from electronic trade on a macroeconomic level. In the short term, the advantages are expected to be concentrated in rich nations, while emerging countries will benefit more in the long run.

E-commerce will boost the amount of international trade. Knowledge spillovers will help nations that are exposed to imports from high-income economies. Furthermore, it is predicted that electronic commerce would both generate and destroy employment (Terzi, 2011). The Internet penetration rate has likewise surpassed two billion and is continuing to rise. The impact of e-commerce is far-reaching. It is most commonly employed as a trade system in which sellers and buyers may agree on a fair market price. For example, eBay is the largest e-commerce market place, having more than 80 million members globally, where anybody can virtually buy and sell. eBay, founded in 1995, brings together a varied and enthusiastic community of individual buyers and sellers and small companies. Their combined influence on e-commerce is enormous. When structured through electronic marketplaces and e-commerce applications, the Internet lowers information costs and allows customers and sellers to be matched and interact electronically, decreasing the importance of geographic proximity and traditional business networks.

Earlier research revealed strong evidence that the growth of global markets via the Internet makes historical ties less significant, and that nations with the fewest prior trade relationships, particularly developing countries, have the most to benefit from the Internet (Freund and Weinhold, 2004). According to the findings based on data from the United States and Europe, electrical parts, food, medicines, and forest/paper goods are the most web demanding industries. It is conceivable that these similar sectors and businesses would be affected by e-commerce by exporting in other locations (Terzi, 2011). Transnational firms, on the other hand, appear to be the most active consumers of Internet trade, according to recent data. Countries that have more interactions with the outside world, whether through commerce, tourism, or geography, are more likely to have modern digital technologies compared to other nations. Keeping in view the impacts of international trade on e-commerce, the following hypothesis was developed to test the significance of the study.

*H_6_: International trade has a positive effect on e-commerce*

**FIGURE 1:**
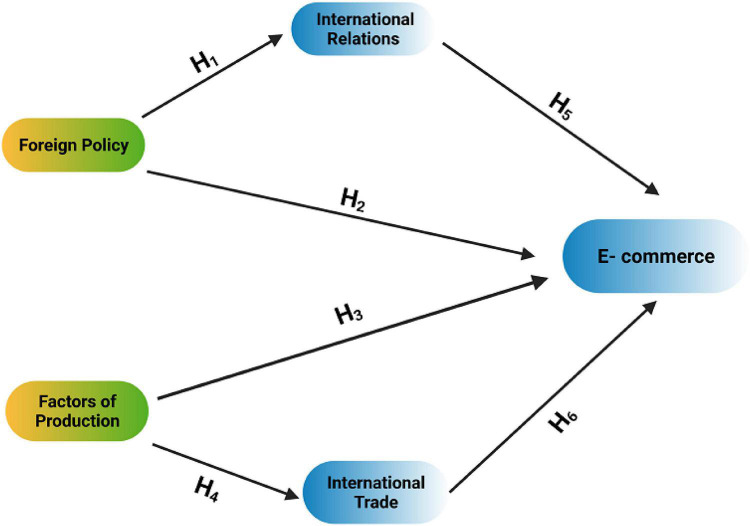
Conceptual model.

Based upon the literature review, this research was designed and the following conceptual framework was developed (see Figure 1). The research revolves around this framework.

## Research Methods

This part of the paper analyzes the hypotheses proposed in the literature review. The main focus of the study was measuring the effects of foreign policy on international relations and consequently on e-commerce. Similarly, the other focus of the study was to find the role of factors of production on e-commerce considering the mediating role of international trade. Of the two major approaches for conducting research, this study followed the quantitative approach which quantifies the variables for better measurement, understanding, and analysis. Since this study measured the effects of certain variables, positivism was the philosophical approach used for measuring variables (Androniceanu et al., 2020). This study used a structured questionnaire for the data collection process, where the scale was adapted from their respective previous studies. The questionnaire was designed on a five-point Likert scale accounting for the disagreement to agreement of the respondent. The data were collected at a single point of time, hence it was a cross-sectional study. The population for this study was the employees of firms working under One Road One Belt. These firms included logistic firms, construction firms, marketing, and engineers. The total sample size used in this study was 329. The sampling design used for this study was convenient random sampling since the availability of respondents was a practical issue in this framework (Richey et al., 2005). Therefore, the data were collected from the respondents as per their convenience and the collection process was self-administered to avoid ambiguity in understanding the items. The total number of completed questionnaires was 329. The data collected were then analyzed for partial least square using structural equation modeling. The software used for this analysis was Smart-PLS 3.3.3.

The demographics of the respondents were analyzed using frequency and percentage. The demographic sheet was designed using gender (male, female), age, education categorized in five categories, and four sectors to which the respondents belonged. The detailed analysis of demography for the respondents is presented in Table 1.

**TABLE 1:** Demographic summary.

Demographic summary	Frequency	%
**Gender**		
Male	245	74.18
Female	84	25.54
**Age**		
<25	32	9.72
25–30	41	12.46
31–40	102	31.00
41–50	114	34.65
50>	40	12.15
**Education**		
Higher secondary	69	20.97
Bachelor	98	29.78
Masters	80	24.31
Doctorate	40	12.15
Others	42	12.76
**Sector type**		
Construction	76	23.10
Logistics	85	25.83
Marketing	26	7.90
Engineering	142	43.16

*N = 329.*

### Instrument Development

The data were collected through a structured questionnaire. The scales for each variable, i.e., foreign policy, factor of production, international trade, international relations, and e-commerce were adapted from previous research (Paster, 2013; Akhmetshin, 2017; Thurik et al., 2019; Ubaydullaev, 2021) that had used particular scales for their studies for data collection. There were five variables in the theoretical framework, each was measured with their respective scales which made a questionnaire containing 28 items in total. The dependent variable of e-commerce was measured with eight items, the mediating variables international trade and international relations with six and four items, respectively. Moreover, the independent variable of foreign policy was measured with four items and factors of production with six items. These items were measures with an interval scale, i.e., Likert scale where 1 = strongly disagree, 2 = disagree, 3 = neutral, 4 = agree, and 5 = strongly agree. Then, the comprehensive questionnaire was checked for reliability and validity of the items. The tests used for questionnaire reliability were Cronbach alpha reliability and composite reliability. The questionnaire was further validated using discriminant and convergent validity. The discriminant validity was checked with factor analysis while the convergent validity was checked with correlations and heterotrait-monotrait ratio (HTMT).

### Data Analysis

**FIGURE 2:**
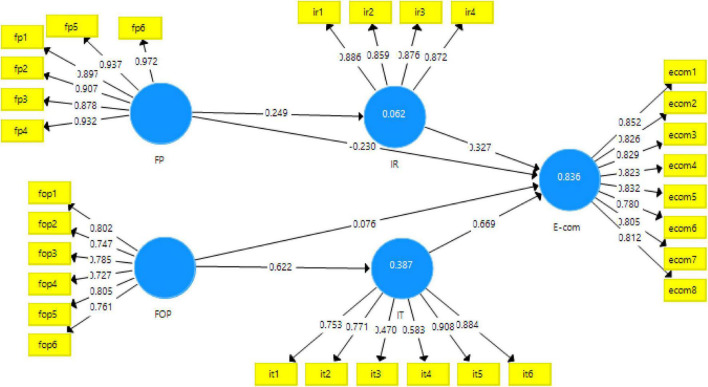
PLS-algorithm for the measurement model.

The data in this study were analyzed with the help of Smart-PLS 3.3.3. Partial least square structural equation modeling was done to get the results. Smart-PLS analyzes the data for structural equation modeling (SEM) in two phases. In the first phase, the data were checked for the measurement model and in the second stage for the structural model. The measurement was checked with the PLS-algorithm and the results produced can be seen in Table 2. In this process, the reliability and validity of the scales was checked. Reliability means that if the data were to be collected again, the same results would be apparent. The reliability in this study was checked with Cronbach alpha and composite reliability. The cut off values for reliability was 0.7. In this case, the reliabilities, i.e., Cronbach alpha (0.833–0.964) and composite reliabilities (0.877–0.971) of all the scales were above this criterion, hence making this scale reliable (Avotra et al., 2021a). Additionally, the validity of the scale was checked with factor loadings of the items for each variable. The maximum threshold stated in literature for factor loadings is 0.6, however, 0.4 is also acceptable (Peterson and Kim, 2013). The values for factor loadings in this study varied from 0.470 to 0.932, therefore, the scale for all the variables confirmed the convergent validity. These can be seen in Table 2 and Figure 2. Also, the average variance extracted (AVE) is said to have a threshold of 0.5. These criteria were fulfilled in this study, therefore the questionnaire was valid.

**TABLE 2:** Measurement model and descriptive statistics.

Constructs	Code	FD	α	CR	AVE	M	SD
**E-commerce**			0.930	0.943	0.673	0.769	0.773
	DE1	0.855					
	DE2	0.827					
	DE3	0.830					
	DE4	0.824					
	DE5	0.833					
	DE6	0.778					
	DE7	0.805					
	DE8	0.808					
**Factors of production**			0.873	0.898	0.595		
	FOP1	0.802					
	FOP2	0.747					
	FOP3	0.785					
	FOP4	0.727					
	FOP5	0.805					
	FOP6	0.761					
**Foreign policy**			0.964	0.971	0.849	0.835	0.835
	FP1	0.897					
	FP2	0.907					
	FP3	0.878					
	FP4	0.932					
	FP5	0.937					
	FP6	0.972					
**International relations**			0.896	0.928	0.763	0.279	0.276
	IR1	0.886					
	IR2	0.859					
	IR3	0.876					
	IR4	0.872					
**International trade**			0.833	0.877	0.555	0.691	0.696
	IT1	0.753					
	IT2	0.771					
	IT3	0.470					
	IT4	0.583					
	IT5	0.908					
	IT6	0.884					

*CR, Construct reliability; AVE, Average variance extracted; α, Cronbach alpha; M, Mean; SD, Standard deviation.*

Validity of the scale, i.e., discriminant validity was confirmed with the help of Fornell and Larcker and the HTMT ratio (heterotrait-monotrait ratio). In Fornell and Larcker, the data show discriminant validity if all the values are less vertically than the top value (Yingfei et al., 2021). This criterion was met in this study as can be seen in Table 3.

**TABLE 3:** Fornell and Larcker criterion.

Variables	E-com	FOP	FP	IR	IT
E-com	**0.820**				
FOP	0.533	**0.772**			
FP	0.197	0.525	**0.921**		
IR	0.813	0.494	0.249	**0.873**	
IT	0.809	0.622	0.457	0.756	**0.745**

*E-com, E-commerce; FP, Foreign policy; IR, International relations; FOP, Factors of production; IT, International trade. Bold values indicate the relationship and significance of test.*

For the heterotrait-monotrait ratio (HTMT) of correlation to show discriminant validity, the values should be less than 0.9 (Peterson and Kim, 2013). This criteria was also met in this study as can be seen in Table 4.

**TABLE 4:** HTMT ratio.

Variables	E-com	FOP	FP	IR	IT
E-com					
FOP	0.514				
FP	0.204	0.661			
IR	0.887	0.493	0.265		
IT	0.901	0.728	0.609	0.832	

*E-com, E-commerce; FP, Foreign policy; IR, International relations; FOP, Factors of production; IT, International trade.*

**FIGURE 3:**
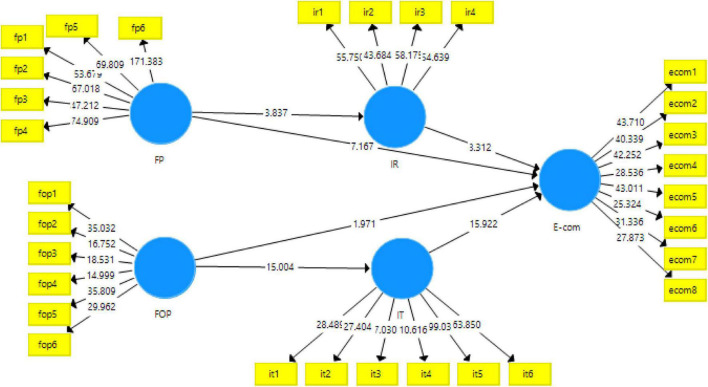
PLS-bootstrapping for the structural model.

In the next step, the data were checked against the hypotheses. For this purpose, this study used the software Smart-PLS 3.3.3. In this step, the data were analyzed for PLS structural equation modeling which is represented with the help of paths. These results show the direct and the indirect effects of the variables. The liner path models were the direct effects, on the other hand, mediations were shown with the indirect effects of the model. The analysis obtained with consistent bootstrapping is explained and shown in Figure 3.

The results obtained for consistent bootstrapping are shown in Table 5. For the hypotheses developed for this study, the data supported seven out of eight. For the first hypothesis, foreign policy plays a very significant role in international relations (*t-statistic* = *3.837; p-value* = *0.000)* counting for 6.2% change in international relations due to the nature of foreign policy. For H_2_, foreign policy could not find a positive effect on e-commerce rather it rejected the hypothesis by showing a negative but significant impact (*t-statistic* = *7.167; p*-value = 0.000). H_3_ and H_4_ were supported by showing a positive and significant impact of factors of production on e-commerce (*t-statistic* = *1.971; p*-value = 0.025^**^) and international trade (*t-statistic* = 15.004; *p-*value = 0.000). However, e-commerce was found to be the most significant and vital variable of the study with *R*^2^ = 83.6% while international trade showed a 38.7% change. Regarding H_5_ and H_6_, they were also supported exhibiting the positive effects of international relations (*t-statistic* = 8.312; *p*-value = 0.000^**^) and international trade (*t-statistic* = 15.922; *p*-value = 0.000^**^) on e-commerce. The summary of the results obtained is given in Table 5.

**TABLE 5:** Results for the structural model.

Paths	H	O	M	*SD*	T-Stats	*P*-Value	*R* ^2^	Results
FP - > IR	H_1_	0.249	0.248	0.065	3.837	0.000***	0.062	Supported
FP - > E-com	H_2_	−0.230	−0.226	0.032	7.167	0.000***		Not Supported
FOP - > E-com	H_3_	0.076	0.076	0.038	1.971	0.025**	0.836	Supported
FOP - > IT	H_4_	0.622	0.624	0.041	15.004	0.000***	0.387	Supported
IR - > E-com	H_5_	0.327	0.329	0.039	8.312	0.000***		Supported
IT - > E-com	H_6_	0.669	0.668	0.042	15.922	0.000***		Supported

Significance level *** = 0.005%, ** = 0.05%. H, Hypothesis; O, Original sample; M, Sample mean; SD, Standard deviation; E-com, E-commerce; FP, Foreign policy; IR, International relations; FOP, Factors of production; IT, International trade.

## Discussion

From the onset of the OBOR initiative, China took gigantic steps toward economic stability and progress. With the start of this initiative, opportunities for cross-border trade and commerce appeared. With the advancement in digital technologies worldwide, it opened doors for e-commerce across the borders of every country. The countries associated with OBOR were also well aware of this change in the world order. This road not only provides logistic support but also opportunities of numerous businesses across 57 countries. Emerging e-commerce companies were able to benefit from this initiative and obtained a firm standing. In this perspective, this research was designed based upon several contributing factors in the form of hypotheses. The foreign policy of China has been far-reaching for the last 20 years. China had foreseen the futuristic insight of the coming world order long ago. They devised their foreign policy according to the need of time. Along with devising the foreign policy, China also worked on bettering its international relations with up to 57 countries. All countries had benefits in associating with the One Belt One Road initiative. A major country which is going to get the most out of it after China is Pakistan. The trade between both countries has also historically flourished. Now the shift in policy is opening new opportunities for e-commerce between these countries. Along with this, international trade plays a decisive role in strengthening business activities. In giving full shape to e-commerce across borders, other major contributors are land, labor, capital, and entrepreneurship. These are the factors of production which provide the framework for success of any business. These also show their impact on e-commerce and are associated with the framework of this study. The research was carried out in China and the respondents were contractors, markets, logistic vendors, and engineers.

This research focused on several hypotheses to analyze the impact of foreign policy on international relations and consequently on e-commerce. Similarly, the other focus of the study was to find the role of factors of production on e-commerce considering the mediating role of international trade. Of the two major approaches for conducting the research, structural equation modeling was carried out using Smart-PLS. A theoretical framework was designed, and questionnaires were sent to the participants. The results supported the hypotheses. The results were also in accordance with many researchers and some were of a different opinion. The possible reasoning for the obtained results is also discussed here. A total of 75% of the respondents were men and 25% were women. They belonged to different professions. The cut off values for reliability was 0.7 (Avotra et al., 2021b). In this case, the reliabilities, i.e., Cronbach alpha (0.833–0.964) and composite reliabilities (0.877–0.971) of all the scales were above this criterion, hence making this scale reliable. The maximum threshold stated in literature for factor loadings is 0.6 (Sarfraz et al., 2021), however, 0.4 is also acceptable (Peterson and Kim, 2013). The possible reason for getting these results was the authenticity and reliability of the data collected from the participants. Discriminant validity was also tested and found satisfactory for the research. This is also due to the authenticity of the data. For the other criterion, i.e., HTMT ration, the researchers agree that the value should not exceed 0.9, i.e., all values should be less than this. In the third phase of data analysis, the data were analyzed for the structural model or path analysis using bootstrapping with Smart-PLS 3.3.3.

This is usually the subsequent stage of the measurement model. The significance of the relationships is usually expressed in the form of path analysis, which either shows the direct effects or the indirect effects. The direct effects are the general linear regression, however, indirect effects indicate the mediating variables. For the first hypothesis, foreign policy plays a significant role in international relations, counting for a 6.2% change in international relations due to the nature of foreign policy. This is due to the fact that foreign policy always plays an important role in devising and molding international relations according to need. For H_2_, foreign policy could not find a positive effect on e-commerce, rather the data rejected the hypothesis by showing a negative but significant impact; this is different to the results of many researchers of the past. These results could be due to the participants of the research having different perspectives about foreign policy. H_3_ and H_4_ were supported by showing a positive and significant impact of factors of production on e-commerce and international trade. These results were in accordance with many other researchers. The possible reason for these results is the reality that factors of production, such as land, labor, capital, and entrepreneurships, are the most important factors for the success of any kind of business.

However, e-commerce was found to be the most significant and vital variable of the study showing *R*^2^ = 83.6%, while international trade showed a 38.7% change. These results indicate that there was strong regression between e-commerce and other variables. Furthermore, H_5_ and H_6_ were also supported exhibiting the positive effects of international relations and international trade on e-commerce. These results also showed accordance to several past researchers (Terzi, 2011). The reason for such results is very simple: that international relations and international trade are the major components of economies. These components can affect any kind of business let alone e-commerce.

## Conclusion

China has excelled in providing the cheapest products around the world. With the advancement in mutual understanding with neighboring countries, OBOR has opened new doors for partnerships around the world for China. This has motivated the manufacturers and foreign policy makers to be more vigilant for reaping the maximum outputs. Not only this but they have also been encouraged to ship their products to the world at the lowest possible prices by cutting down transportation and logistics costs. Also, more cheap labor will be utilized through this project. This study has found that international trade is impacted by the cheaper factors of production that consequently delay e-commerce from reaching the rest of the world. Similarly, foreign policy and international relations have also been found to be the key players for e-commerce. The study has contributed to the literature by finding the significance of OBOR for manufacturers, establishments, and trade and commerce personnel. This research has several implications for future researchers and e-commerce players who are interested in repeating this research with their available resources in different regions. These can be exploited well in finding new avenues for certain research like this.

## Data Availability Statement

The original contributions presented in the study are included in the article/supplementary material, further inquiries can be directed to the corresponding author/s.

## Ethics Statement

All subjects gave their informed consent for inclusion before they participated in the study. The study was conducted in accordance with the Declaration of Helsinki, and the protocol was approved by the Jiangxi University of Science and Technology (JUST), China.

## Author Contributions

HW conceived and designed the concept and wrote the manuscript. BL collected the data and provided technical support. Both authors have read and agreed to the published version of the manuscript.

## Conflict of Interest

The authors declare that the research was conducted in the absence of any commercial or financial relationships that could be construed as a potential conflict of interest.

## Publisher’s Note

All claims expressed in this article are solely those of the authors and do not necessarily represent those of their affiliated organizations, or those of the publisher, the editors and the reviewers. Any product that may be evaluated in this article, or claim that may be made by its manufacturer, is not guaranteed or endorsed by the publisher.
